# Differential Effects of Physical and Social Enriched Environment on Angiogenesis in Male Rats After Cerebral Ischemia/Reperfusion Injury

**DOI:** 10.3389/fnhum.2021.622911

**Published:** 2021-03-26

**Authors:** Xin Zhang, Jing-Ying Liu, Wei-Jing Liao, Xiu-Ping Chen

**Affiliations:** ^1^Department of Rehabilitation Medicine, Zhongnan Hospital of Wuhan University, Wuhan, China; ^2^Department of Rehabilitation Medicine, The first Affiliated Hospital of Nanchang University, Nanchang, China

**Keywords:** ischemia/reperfusion, enriched environment, angiogenesis, VEGF, ANG-1, Tie-2

## Abstract

Different housing conditions, including housing space and the physiological and social environment, may affect rodent behavior. Here, we examined the effects of different housing conditions on post-stroke angiogenesis and functional recovery to clarify the ambiguity about environmental enrichment and its components. Male rats in the model groups underwent right middle cerebral artery occlusion (MCAO) followed by reperfusion. The MCAO rats were divided into four groups: the physical enrichment (PE) group, the social enrichment (SE) group, the combined physical and social enrichment (PSE) group and the ischemia/reperfusion + standard conditioning (IS) group. The rats in the sham surgery (SS) group were housed under standard conditions. In a set of behavioral tests, including the modified Neurological Severity Score (mNSS), rotarod test, and adhesive removal test, we demonstrated that the animals in the enriched condition groups exhibited significantly improved neurological functions compared to those in the standard housing group. Smaller infarction volumes were observed in the animals of the PSE group by MRI detection. The enriched conditions increased the microvessel density (MVD) in the ischemic boundary zone, as revealed by CD31 immunofluorescent staining. The immunochemical and q-PCR results further showed that environmental enrichment increased the expression levels of angiogenic factors after ischemia/reperfusion injury. Our data suggest that all three enrichment conditions promoted enhanced angiogenesis and functional recovery after ischemia/reperfusion injury compared to the standard housing, while only exposure to the combination of both physical and social enrichment yielded optimal benefits.

## Introduction

An enriched environment (EE) classically consists of a large amount of space, social enrichment (SE), and diverse multisensory stimulation, which are supposed to facilitate enhanced sensory, cognitive, motor, and social stimulation compared to those provided by standard laboratory housing conditions (Hannan, 2014). EE provides animals with optimal conditions for enhanced exploration, cognitive activity, social interaction, and physical exercise (Wadowska et al., 2015). Early studies have demonstrated that EE treatment elicits many positive effects at the molecular, anatomical, and functional levels after brain injury (Cassarino and Setti, 2015). However, despite the largely positive evidence, non-significant or even negative effects of EE have also been reported (Fuss et al., 2010; Walker and Mason, 2011). The reason for these inconsistent results could be due to the variety in EE settings. Given that EE is a comparative concept and includes several key components (social interaction, cognitive activity and physical exercise), one important question that arises from EE studies is the extent to which the different components of EE can be separated and analyzed with respect to their beneficial effects. The most studied aspect has been physical exercise. In animal experiments, there are two kinds of exercise interventions: voluntary and forced (Luo et al., 2007; Zhang et al., 2010). Many studies have shown that increased voluntary physical exercise can enhance cognition and alter motor states in wild-type rodents and may induce some cellular changes after stroke (Pang and Hannan, 2013; Voss et al., 2013). Grégoire et al. (2014) confirmed the central importance of exercise in EE when they evaluated the impacts of individual variables of EE on adult hippocampal neurogenesis in healthy adult male CD1 mice. However, Johansson and Ohlsson (1996) reported that the effects of physical enrichment (PE) (exercise) in the form of wheel running on motor performance were inferior to those of social interaction. They also confirmed that an EE allowing free physical activity combined with social interaction resulted in the best performance in ischemic rats. The PE condition in this study added a number of complex auditory, olfactory, visual, and tactile stimuli for the animals, which were intended to induce voluntary exercise. The SE condition provided the animals with more social interaction and relatively more sensorimotor stimulation by housing more animals together in a larger space. Social enrichment has also been proven to have a beneficial effect on neurogenesis (Venna et al., 2014), while social isolation has adverse effects on cognition (Fone and Porkess, 2008). Previous animal studies also indicated that social stimulation is an independent factor that promotes beneficial effects on the central nervous system by reducing anxiety-like behaviors, stimulating voluntary exercise, promoting neurogenesis and facilitating functional recovery following stroke (Fowler et al., 2002; Silasi et al., 2008; Cirulli et al., 2010; Xie et al., 2013). From a theoretical perspective, Will and associates (Will et al., 2004) argued that the social component of the EE paradigm increased exploration and locomotor activity, as the rats “play” in an interactive milieu. Diverse EE paradigms are used between laboratories, with little understanding of how individual EE variables (such as social interactions and physical exercise) might differentially impact specific downstream biological mechanisms.

Angiogenesis is a form of neurovascular remodeling of great importance in numerous pathological conditions, including stroke. Post-stroke angiogenesis not only increases collateral circulation and restores oxygen and nutrient supply to the injured tissue but also provides neurotrophic support to concurrent neurogenesis and synaptogenesis, which all ultimately lead to long-term functional recovery (Petcu et al., 2010). Substantial evidence has shown that neurogenesis and angiogenesis after ischemic stroke are coupled processes and should be acknowledged and pursued as concurrent and non-mutually exclusive events to develop further neurorestorative therapy strategies (Font et al., 2010; Lacar et al., 2012; Ruan et al., 2015). A previous study showed that voluntary physical activity improves long-term stroke outcome in wild-type mice and that this effect was related to augmentation of angiogenesis and cerebral blood flow within the ischemic striatum (Gertz et al., 2006). Our previous research as well as other studies have demonstrated that typical EE enhanced angiogenesis and reduced neurologic deficits in ischemic rats (Seo et al., 2013; Yu et al., 2014; Zhang et al., 2017). However, the housing paradigms in these studies could not unambiguously evaluate the effects of separate EE variables, such as social interaction and physical exercise, which were also reported to influence angiogenesis and neurogenesis (Leasure and Decker, 2009; Zhang et al., 2013). Clearly defining the relative and/or combinatorial effects of such variables is essential for the design of EE paradigms for both research and rehabilitative use.

In this study, we established a set of different housing conditions to experimentally isolate the social interaction and physical exercise provided by typical EE with the aim of gaining insights into their isolated and combined contributions to functional recovery and angiogenesis after stroke.

## Materials and Methods

### Animals

**FIGURE 1:**
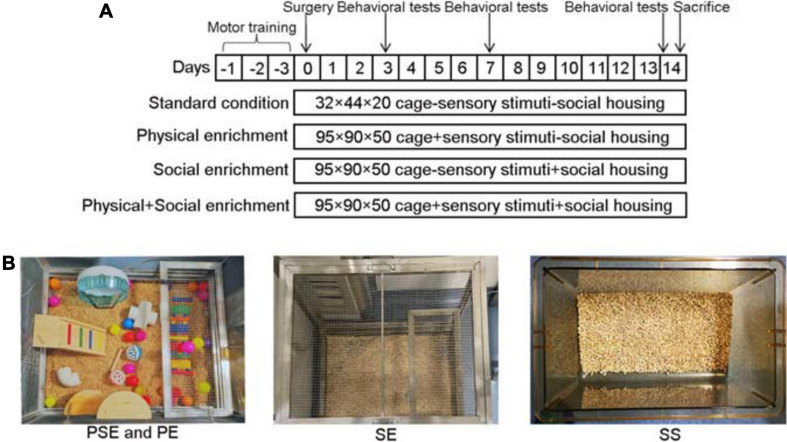
Flow chart of the experimental protocol and enrichment settings. **(A)** The experimental protocol and group settings. **(B)** The setting of PSE, PE, SE, and SS.

A total of 78 healthy adult male Sprague–Dawley rats (purchased from the Experimental Animal Center of Wuhan University, Wuhan, Hubei, China) weighing 220–240 g were housed in a controlled environment (20 ± 1°C, 55 ± 5% relative humidity, light period from 8:00 to 20:00) with free access to fresh water and standard rat chow. In order to exclude the effect of gender on stroke recovery, only male rats were selected for the study (Koellhoffer and McCullough, 2013). After arrival, all rats underwent 3 days of beam-walking, rotarod, and adhesive removal training to prepare for future neurological function tests. Following training, the animals were numbered and randomized into different groups by using a table of random numbers. Then the rats were randomly assigned to one of the following groups: the ischemia/reperfusion (I/R) + SE group (*n* = 16), the ischemia/reperfusion + PE group (*n* = 16), the ischemia/reperfusion + physical and social enrichment group (PSE, *n* = 16), the ischemia/reperfusion + standard condition group (IS, *n* = 16) and the sham + standard condition group (SS, *n* = 14). The experimental protocol and group settings are shown in Figure 1A. All animal experimental procedures were approved according to the animal experimental committee of Wuhan University at Wuhan, China. Every effort was made to minimize the number of animals used and their suffering. Animals were anesthetized with inspired concentrations of 4% (induced) and 2% (maintained) isoflurane. The brains were transcranial perfused with 200 mL of 0.9% saline followed by perfusion and immersion in 4% paraformaldehyde solution. The excluded animals were euthanized with inhalation of CO2. There were no surviving animals at the end of study.

### Surgery

Occlusion of the right middle cerebral artery was performed using the modified intraluminal filament technique. All procedures were performed according to the Guidelines of the National Institutes of Health Guide for the Care and Use of Laboratory Animals. Surgical anesthesia was induced and maintained with inspired isoflurane at concentrations of 4 and 2%, respectively, in 2:1 N_2_O:O_2_. The body temperature was maintained at 37.0 ± 0.5°C with warming pads. After a 2 cm median incision of the neck skin, the right carotid artery (CCA), external carotid artery (ECA), and internal carotid artery (ICA) were carefully isolated. After that, a monofilament nylon filament (Beijing Cinontech Biotech Co., Ltd., Beijing, China) was inserted through the right CCA and gently advanced into the ICA to a point approximately 18 mm distal to the bifurcation of the carotid artery, and the right MCA was then occluded. After 2 h, the filament was gently withdrawn for reperfusion. In the sham surgery (SS) group, all surgical procedures were the same except a nylon filament was not inserted (Longa et al., 1989). While the time of SS was 3 min shorter on average than that of the surgery group, no difference was found in the total anesthesia duration between sham animals and I/R animals. Three rats did not survive the surgery due to excessive bleeding because the vagus nerve was accidentally cut while separating it from the ICA.

### Acute Neurological Evaluation

After the rats recovered from anesthesia, the neurologic deficit findings were scored on a five-point scale to determine whether the middle cerebral artery occlusion (MCAO) model was successfully induced (Longa et al., 1989). Rats with scores of 1–3 were considered successful models and were included in the study. Six rats with scores of 0, which indicated no neurologic deficit, and four rats with scores of 4, which indicated a depressed level of consciousness, were excluded. Five rats were excluded during the 2-week follow-up due to cervical hematoma compression or subarachnoid hemorrhage; thus, the ultimate number of rats in each group was 12. All of the excluded animals were euthanized by CO2 inhalation to minimize their suffering, and the procedures were approved according to the Institutional Animal Care and Use Committee of Wuhan University.

### Housing Conditions

One day after MCAO, rats of different groups were returned to their corresponding cages. The rats in the PE, SE, and PSE groups were kept in the corresponding environments for 4 h in the morning and were kept under standard conditions for the rest of the experiment. The rats in the SE groups were always grouped with the same rats in the social EE. The rats in the IS and SS groups received continuous standard housing. Additionally, the IS and SS rats were moved for 4 h from their usual standard cage to a different standard cage to equalize the handling procedures of all rats. The details of the different conditions were as follows:

#### Standard Conditions

The rats were housed in standard laboratory cages that were 32 cm wide × 44 cm long × 20 cm high with nothing but bedding materials inside. The rats were housed in groups of two.

#### Socially Enriched Environment

Animals were housed in a 75 cm wide × 90 cm long × 50 cm high stainless-steel network surface cage with bedding materials inside. Six to ten rats were housed together in one cage. And they were always grouped with the same rats in the social EE.

#### Physically Enriched Environment

Animals were housed in a 75 cm wide × 90 cm long × 50 cm high stainless-steel network surface cage with bedding materials, several differently-colored plastic balls, differently-shaped wooden blocks, a T-shaped and an L-shaped plastic tunnel, climbing ladders and a running wheel inside for exploration and exercise. Three times a week, the plastic balls and wooden blocks were changed with new items of different colors or shapes, and the tunnels, ladders, and running wheel were relocated. The rats were housed in groups of two.

#### Physically and Socially Enriched Environment

The cage and objects were the same as the physically EE, while the rats were housed in groups of 6–10. The water and food supply were the same for all animals (Figure 1B).

### Neurological Function Tests

A set of behavior tests, including the Modified Neurological Severity Score (mNSS), rotarod test and adhesive removal test, were carried out at 3, 7, and 14 days after MCAO by an investigator who was unaware of the experimental groups.

The mNSS was scored on a scale of 0–18 (no deficit score, 0; maximal deficit score, 18) and included a composite of tests to evaluate motor (muscle status, abnormal movement), sensory (visual, tactile, and proprioceptive), reflex, and balance abilities. One point was scored for the failure to perform a certain task or the absence of a particular reflex. Thus, a higher score indicated a more severe injury (Jin et al., 2014).

A rotarod test with accelerating velocity was employed to measure balance and coordination deficits of rats (LE8200 Panlab, Harvard Apparatus, United States). During the test, accelerating velocity (4–40 rpm) was achieved within 260 s with a maximal testing time of 300 s. The time until the animals dropped was measured. Tests were always performed twice, and the means were used for statistical analysis (Doeppner et al., 2014).

The adhesive removal test was performed to measure somatosensory deficits. Briefly, two small pieces of adhesive-backed paper dots (of equal size, 113.1 mm^2^) were used as bilateral tactile stimuli occupying the distal-radial region on the wrist of each forelimb. The time required to remove the stimulus from each limb was recorded. Each animal received five trials at 3, 7, and 14 days after surgery, and the mean time required to remove both stimuli from the limbs was recorded (Diederich et al., 2014).

### Magnetic Resonance Imaging for Infarct Volume

We used a Bruker 7.0T magnetic resonance imaging (MRI) horizontal scanner (Institute of Physics and Mathematics, Chinese Academy of Sciences, Wuhan, China) to quantify the lesion volume at 14 days after MCAO (*n* = 6/group). The rats were anesthetized with inspired concentrations of 4% (induced) and 2% (maintained) isoflurane. Coronal images were obtained with the central slice 0.5 mm from bregma. T2-weighted multislice images were acquired using a RARE sequence with the following parameters: time-to-repetition (TR) = 3,000 ms, effective time-to-echo = 36 ms, RARE factor = 8, matrix size = 256 × 256, field-of-view = 30 mm × 30 mm, and BR = 24 slices with a slice thickness of 0.8 mm. For further processing, MRI sequences were exported as DICOM sequences. The volume of ischemic lesions was determined by a blinded investigator using ImageJ software (NIH, Bethesda, MD, United States). The total infarct area was obtained by adding the infarct areas of all sections and multiplying the value by the slice thickness to derive the infarct volume. The percentage of infarct volume was calculated by the following equation: percentage of infarct volume = (infarct volume/contralateral hemispheric volume) × 100%.

### Immunofluorescent Staining

Brain paraffin sections (4 μm) were performed heat mediated antigen retrieval with Tris/EDTA buffer for 20 min after hydrated. The sections were treated with 0.1% Triton-X for 10 min and blocked with 10% FBS for 1 h and then primarily incubated with mouse monoclonal to CD31 antibody (1:100, Abcam, Cambridge, United Kingdom; product code: ab-119339, RRID: AB_10936456; Immunogen: Human CD31) at 4°C overnight. After washing, the brain sections were then incubated with Cy3-conjugated goat anti-mouse IgG secondary antibody (1:50, Jackson ImmunoResearch Labs, West Grove, PA, United States; Product Code: 115-166-003, RRID: AB_2338699) for 1 h. For quantitative measurements of microvessel density (MVD), five slides from each brain, with each slide containing four fields (× 200) from the penumbra, were captured under a fluorescence microscope (Olympus Corporation, Tokyo, Japan) and analyzed using ImageJ software (NIH, Bethesda, MD, United States). The interval between the sections was 200 μm. Ischemic boundary zone was defined as a region of 300 μm distal to the stroke core with hypovascularization (Figure 4C; Rust et al., 2019). Any endothelial cell or group of cells that stained positive for CD31 and was distinct from neighboring tissues was counted as a microvessel. The average of the values obtained by the two reviewers for each field was reported as a single numerical value, and the mean count from the five regions was used to determine the MVD score.

### Immunohistochemical Assessment

Rats (*n* = 6/group) were killed at 14 days after MCAO. The brains were transcardially perfused with 200 mL of 0.9% saline followed by perfusion and immersion in 4% paraformaldehyde solution before being embedded in paraffin. A series of 4 μm-thick brain sections were sliced from the paraffin block for immunohistochemistry to detect the expression of angiopoietin-1 (Ang1) and its receptor Tie-2. The procedure was performed as reported previously (Cui et al., 2009). Brain sections were hydrated and performed heat mediated antigen retrieval with Tris/EDTA buffer for 20 min before commencing with IHC staining protocol. The slices were then treated with 3% H_2_O_2_ for 10 min and blocked with 1% BSA for 1 h. And the samples were incubated overnight at 4°C with a rabbit polyclonal antibody against angiopoietin 1 (1:100, Abcam, Cambridge, United Kingdom; Product Code: ab-102015, RRID: AB_10712377; Immunogen: Synthetic peptide corresponding to human angiopoietin 1 internal sequence aa 225–239), rabbit polyclonal antibody against Tie-2 (1:100, Santa Cruz Biotechnology, Inc., Dallas, TX, United States; Product Code: sc-9026, RRID: AB_2203226; Immunogen: Extracellular domain of human Tie-2 aa 25–200). After three rinses (5 min each) with phosphate-buffered saline (PBS, pH = 7.4), the sections were reacted with peroxidase-labeled anti-rabbit IgG (1:200, Dako, Agilent Pathology Solutions, Santa Clara, CA, United States; Product Code: K4003, RRID: AB_2630375). Negative controls were established by replacing the primary antibody with PBS. For qualitative analysis, four visual fields (×200) from each section in the penumbra were captured under a light microscope (Olympus Corporation, Tokyo, Japan).

### Real-Time Quantitative (q)-and Reverse Transcription (RT)-PCR

Rats (*n* = 6/group) were decapitated after MRI examination, and cortical tissue samples were collected. qRT-PCR was performed to evaluate vascular endothelial growth factor (VEGF) gene expression. Total RNA was extracted from the cerebral cortex of the penumbra using TRIzol (Invitrogen, United States). Adding trichloromethane into the homogenate. The mixture was centrifuged at 10,000 *g* at 4°C for 10 min. Isopropanol precooled at an equal volume of 4°C was added, mixed upside down, and stood at −20°C for 15 min. Then the solution was centrifuged at 10,000 *g* at 4°C for 10 min. Pre-cooled 75% ethanol was added at 4°C and reversed for several times. The RNA was washed and centrifuged at 10,000 *g* at 5 min. After drying for several minutes, ethanol was fully volatilized and RNA was fully dissolved in Rnase-free Water. Total RNA was reverse transcribed using a First Strand cDNA Synthesis Kit (Toyobo, Japan). Real-time PCR was performed using SYBR^®^ Premix Ex Taq^TM^ Mix (TaKaRa, Japan) in a StepOne^TM^ Real-Time PCR System (Life Technologies). The following PCR conditions were used: 95°C for 1 min, followed by 40 cycles of 95°C for 15 s, 58°C for 20 s, and 72°C for 45 s. The forward and reverse primer sequences used for real-time PCR were as follows:

VEGF (F): 5′-TGTGAGCCTTGTTCAGAGCG-3′;
VEGF (R): 5′-GACGGTGACGATGGTGGTGT-3′;
Ang-1(F): 5′-TAACCTCGCCCTGCAAAGAG-3′;
Ang-1(R): 5′-CTGTATGCTTGCAGGTGGTGAT-3′;
GAPDH (F): 5′-CGCTAACATCAAATGGGGTG-3′;
GAPDH (R): 5′-TTGCTGACAATCTTGAGGGAG-3′.

### Statistical Analysis

Statistical analyses were performed using SPSS 20.0 statistical software. The behavioral test scores were analyzed with conventional two-factor analyses of variance (ANOVA) followed by Bonferroni’s *post hoc* tests. The other data were analyzed using one-way ANOVA followed by Bonferroni’s *post hoc* tests. All data were normally distributed and are presented as the mean ± standard deviation (SD). Normal distribution was evaluated by the Kolmogorov–Smirnov test. A value of *P* < 0.05 was considered significant.

## Results

### Enriched Conditioning Improved Neurological Functional Outcomes

**FIGURE 2:**
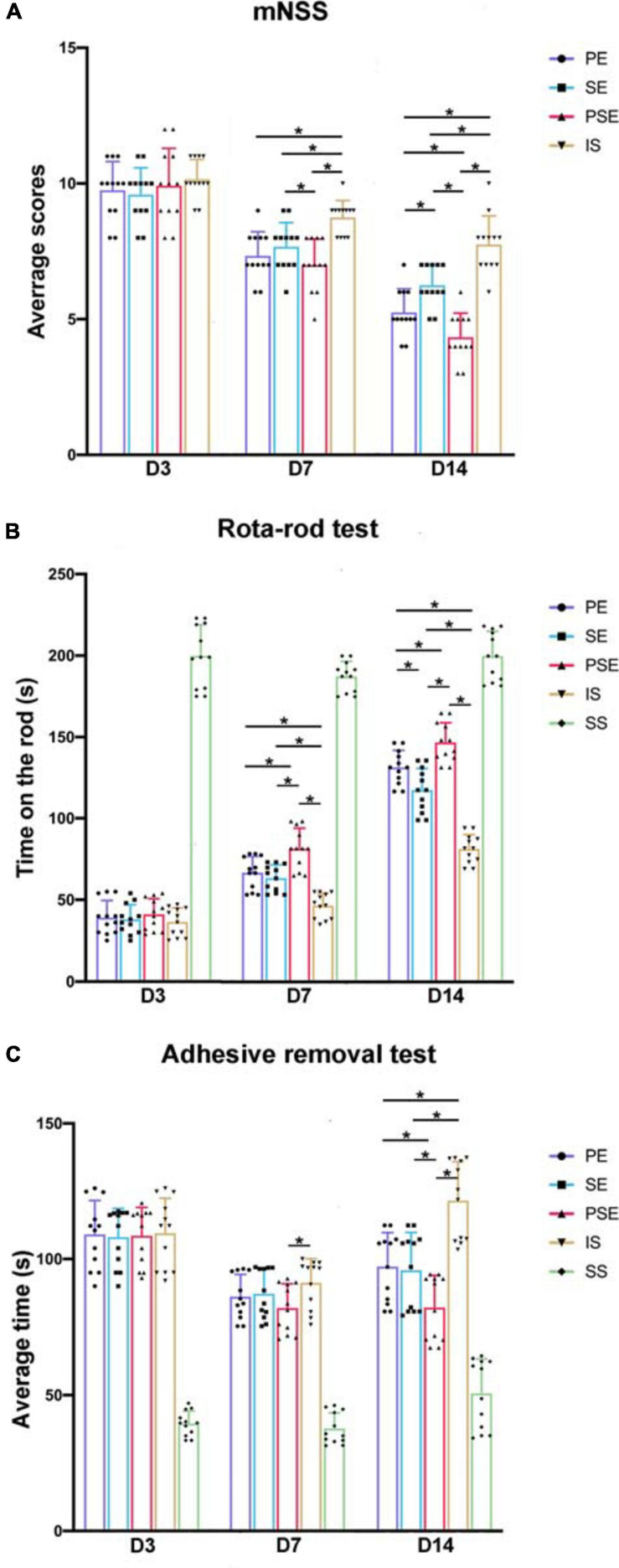
Behavioral tests. The results of the modified Neurological Severity Score (mNSS) **(A)** rota-rod test **(B)** and adhesive removal test **(C)** on 3, 7, and 14 days of PE, SE, PSE, IS, and SS group. *n* = 12. Data are expressed as mean ± SEM; *P < 0.05.

We first compared the effects of different housing conditions on motor deficits using the mNSS, rotarod test and adhesive removal test at 3, 7, and 14 days after MCAO. Two-way ANOVA revealed significant differences due to time [*F*_(2,88)_ = 287.65, *n* = 60, *P* < 0.001], group [*F*_(4,88)_ = 475.96, *n* = 60, *P* < 0.001], and the interaction between these two factors [*F*_(8,88)_ = 26.19, *n* = 60, *P* < 0.001] in the mNSS results. Rats in the sham + standard condition group had mild neurological symptoms but fully recovered 3 days after the SS. There were no significant differences in mNSS results among the ischemia/reperfusion groups (the ischemia/reperfusion + PE group, the ischemia/reperfusion + SE group, the ischemia/reperfusion + PSE group and the ischemia/reperfusion + standard condition group) on day 3 (*P* > 0.05). The ischemia/reperfusion rats exhibited progressive functional recovery on day 7, but the ischemia/reperfusion + PE group, the ischemia/reperfusion + SE group and the ischemia/reperfusion + PSE group exhibited better recovery than the ischemia/reperfusion + standard condition group (*P* < 0.001 vs. PE,*P* = 0.001 vs. SE,*P* < 0.001 vs. PSE). On day 14, the mNSS scores of the three enriched conditioning groups were significantly lower than those of the ischemia/reperfusion + standard condition group (*P* < 0.001). The rats in the ischemia/reperfusion + PE group showed a much better functional recovery than those in the ischemia/reperfusion + SE group (*P* = 0.003), while the ischemia/reperfusion + PSE group rats achieved the best recovery among the three enriched groups (*P* = 0.007 vs. PE, *P* < 0.001 vs. SE, Figure 2A).

For the rotarod test, there were significant differences due to group [*F*_(4,88)_ = 901.65, *n* = 60, *P* < 0.001], time [*F*_(2,88)_ = 760.55, *n* = 60, *P* < 0.001], and the group × time interaction [*F*_(8,88)_ = 30.10, *n* = 60, *P* < 0.001]. Rats under enriched conditions exhibited significantly increased time on the rod at 7 and 14 days after MCAO compared to those under standard conditions (*P* < 0.001), while rats in both the ischemia/reperfusion + PSE group and the ischemia/reperfusion + PE group showed a much better functional status than those in the ischemia/reperfusion + SE group at day 14 (*P* < 0.001 vs. *PSE*, *P* = 0.006 vs. *PE*, Figure 2B).

For the adhesive removal test, significant differences were seen due to time [*F*_(4,88)_ = 261.93, *n* = 60, *P* < 0.001], group [*F*_(4,88)_ = 174.69, *n* = 60, *P* < 0.001], and the interaction between these two factors [*F*_(4,88)_ = 15.15, *n* = 60, *P* < 0.001]. At day 7 after MCAO, only the PSE condition led to significantly decreased time required to remove the stimuli compared to the standard condition (*P* = 0.008). At day 14, the rats in the three enriched groups exhibited better functional results than the rats in the ischemia/reperfusion + standard condition group (*P* < 0.001), while the effect of the PSE condition outweighed those of the PE and the SE conditions (*P* = 0.007 vs. *PE, P* = 0.013 vs. *SE*, Figure 2C).

### Physical Enrichment and the Combination of Physical and Social Enrichment Reduced Infarct Volume in Ischemia/Reperfusion Rats

**FIGURE 3:**
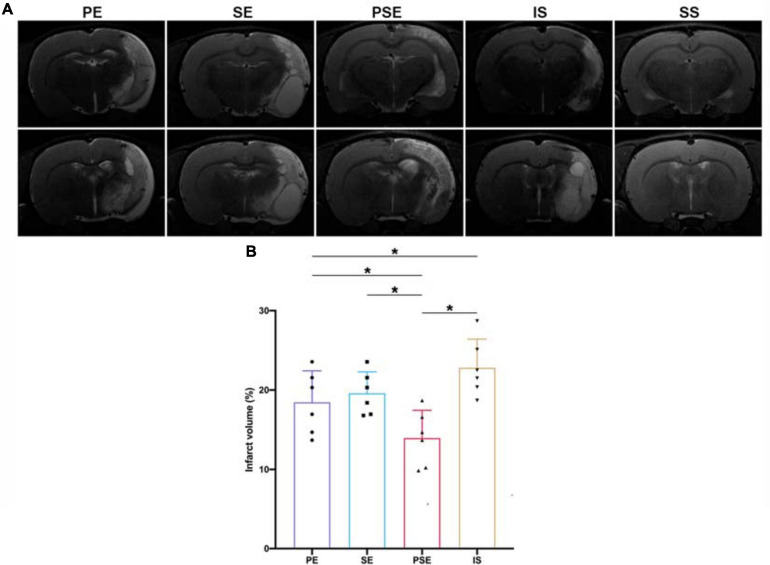
Infarct volumes. **(A)** Representative images of different groups in T2 MRI scans at 14 days after stroke. Bregma levels were –2.8 mm (the upper row) and –5.2 mm (the lower row). **(B)** Quantitative analysis of infarct volumes according to T2 scans in four groups. *n* = 6. Data are expressed as mean ± SEM; *P < 0.05.

To elucidate whether enhanced neurological recovery was a consequence of structural neuroprotection, we analyzed infarct volumes 14 days after stroke. As demonstrated by MRI images (Figure 3A), there was a significant difference among different housing conditions in infarct size [*F*_(3,20)_ = 10.80, *n* = 24, *P* = 0.0002] (Figure 3B). At day 14 after MCAO, the PSE conditioning and the PE conditioning significantly reduced the infarct volume compared to that of the ischemia/reperfusion + standard condition group (*P* < 0.001 vs. *PSE*, *P* = 0.042 vs. PE), while the ischemia/reperfusion (I/R) + SE group exhibited trends of reducing the infarct size, but the effect was not significant (*P* = 0.124) (Figure 3B). These results demonstrated that the neuroprotective effect of reducing infarct volume after MCAO can not be achieved by SE alone.

### Enriched Conditioning Increased Microvessel Density

**FIGURE 4:**
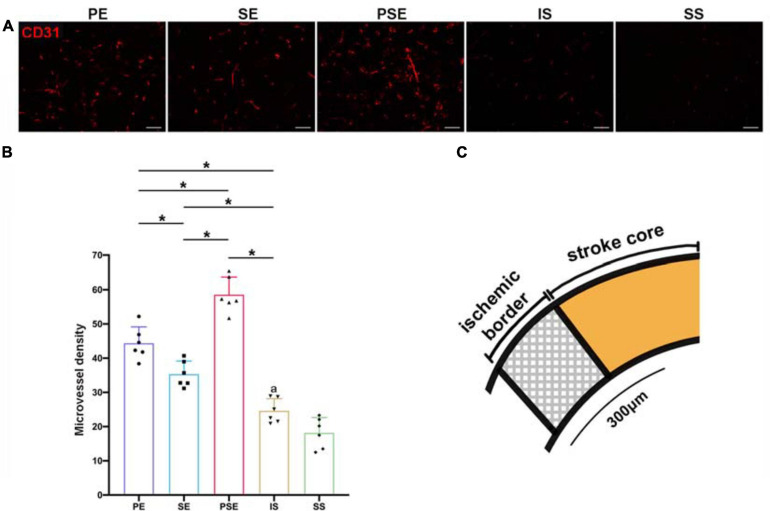
Microvessel density (MVD) in the peri-infarct cortex at 14 days after MCAO. **(A)** Representative images of immunofluorescent labeling of CD31 in the periinfarct area. Bar = 50 μm. **(B)** Quantitative analysis of microvessel density (MVD). *n* = 6. Data are expressed as mean ± SEM; *P < 0.05. **(C)** Schematic brain with a highlight of ischemic border.

To evaluate how different housing conditions affected post-stroke angiogenesis, immunofluorescent staining of CD31, which is expressed in all cells within the vascular compartment and plays diverse roles in angiogenesis, platelet function, and thrombosis (Baumann et al., 2004), was employed. In all ischemia/reperfusion groups, many blood vessels were intensely stained by the CD31 monoclonal antibody around the ischemic region (Figure 4A). Quantitative analysis showed that the MVD of the rats was significantly affected by group [*F*_(4,25)_ = 79.70, *n* = 30, *P* < 0.001]. The enriched housing conditions significantly increased the MVD of the rats compared to that of the rats in the standard housing group. The PSE condition produced a better result than the PE or SE condition alone (*P* < 0.001), while the PE condition showed substantially better results than the SE condition (*P* = 0.002, Figure 4B).

### Enriched Conditions Upregulated the Expression of Angiogenic Factors

**FIGURE 5:**
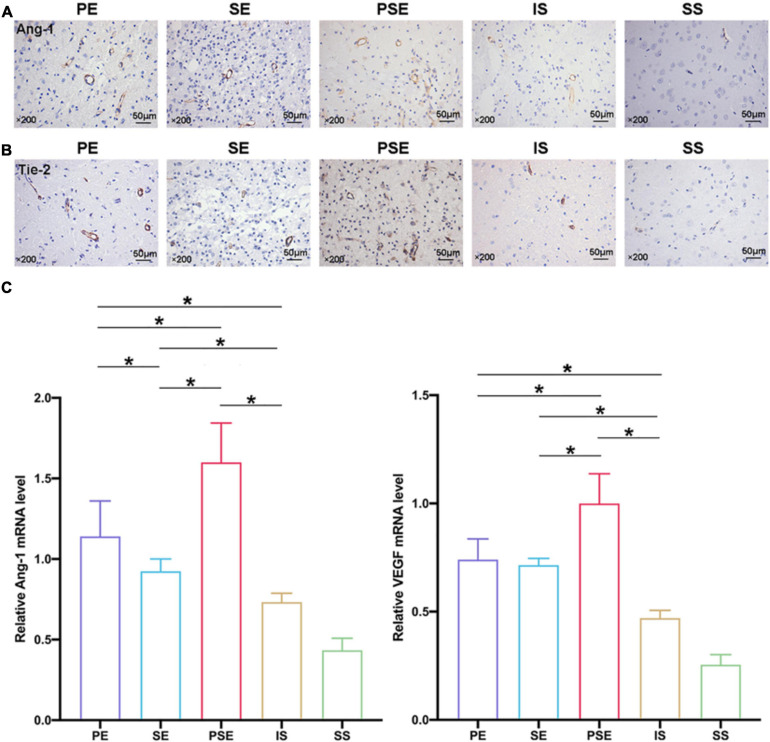
Angiogenic factors expression in the penumbra at 14 days after ischemia/reperfusion. **(A)** Representative magnification images of Ang-1 immunohistochemical staining of five groups. **(B)** Representative images of Tie-2 immunohistochemical staining of five groups. **(C)** Quantitative analysis of relative VEGF/Ang-1 mRNA levels in five groups, while PE, SE, and PSE conditionings increased Ang-1 and VEGF mRNA levels compared to IS group. *n* = 6. Data are expressed as mean ± SEM; *P < 0.05.

To identify the signaling pathways responsible for the enhanced angiogenesis, the expression of the growth factors VEGF and Ang-1/Tie-2, which are two types of vascular regulatory molecules crucial for vessel formation and maturation (Augustin et al., 2009), was analyzed by immunohistochemistry (Figures 5A,B), and q-PCR (Figure 5C) in the penumbra at 14 days after stroke. The immunochemical results showed that the levels of Ang-1 and Tie-2 were significantly higher in the ischemia/reperfusion groups than in the sham + standard condition group. The PE, SE, and PSE conditions increased Ang-1/Tie-2 expression compared to that in the ischemia/reperfusion + standard condition group (Figures 5A,B). The rats in the ischemia/reperfusion + PSE group had significantly higher Ang-1/Tie-2 levels than those in the PE and SE groups. Moreover, the PE condition also had a larger effect on upregulating Ang-1/Tie-2 expression than the SE condition.

The VEGF and Ang-1 mRNA expression levels were further examined by qRT-PCR at 14 days after MCAO. As shown in Figure 5C, **the VEGF** and **Ang**-1 mRNA levels showed the same trends among the five groups, with statistical results of [*F*_(4,25)_ = 115.78, *n* = 30, *P* < 0.001] and [*F*_(4,25)_ = 66.91, *n* = 30, *P* < 0.001], respectively. The rats in the ischemia/reperfusion + PE group, the ischemia/reperfusion + SE group and the ischemia/reperfusion + PSE group exhibited higher VEGF and Ang-1 expression than the rats in the ischemia/reperfusion + standard condition group (*P* < 0.01). The rats in the ischemia/reperfusion + PSE group had significantly higher VEGF/Ang-1 mRNA levels than those in the PE and SE groups, while the rats in the PE group had higher Ang-1 mRNA levels than those in the SE group (*P* < 0.001).

## Discussion

The model used was that of MCAO and Reperfusion. If the reperfusion was not performed, not many of the experimental animals would have survived till 14 days. Given the widely recognized effectiveness and complicated paradigms of EE treatment, the current study constructed different housing conditions for I/R rats with the aim of determining whether a single or specific component of EE is sufficient to confer its neuroprotective benefits or if all components are necessary. The data suggest that the PE, SE, and PSE conditions demonstrated more advantages in promoting functional recovery and angiogenesis than the condition with no enrichment, but only exposure to the PSE condition yielded optimal benefits. This result is in accordance with previous research using a rat model of traumatic brain injury carried out by Sozda et al. (2010). However, the PE condition in the present study exhibited a larger effect on promoting neurological recovery than the SE condition, which is different from the results of the former study (Johansson and Ohlsson, 1996). This contradiction may be due to the different settings in these two studies: in the study by Johansson and Ohlsson, the rats in the PE group were housed individually with a running wheel, while the PE setting in the present study included much more stimulation.

### Intervention Duration

In this study, the intervention time in the PE, SE, and PSE conditions was 4 h instead of 24 h, which was out of consideration of the limitations of clinical treatment. It was reported that 6-h EE produced motor and cognitive benefits on traumatic brain injury similar to those of continuous EE (de Witt et al., 2011). Gaulke et al. (2005) reported that 1 h of EE per day was sufficient to augment cognitive recovery after fluid percussion brain injury. The study by Nygren and Wieloch (2005) showed that short (3 h) daily exposure was preferable to continued housing in EE because there was equal recovery of function and lower mortality. The disparity in the amount of EE necessary to confer functional recovery may be due to the different models of injury along with the diverse training protocols. In this study, 4-h exposure to enriched conditions exhibited better motor results after MCAO than the standard condition. The findings may have clinical relevance: in an inpatient setting, the cognitive and physical stimulation provided by multidisciplinary therapy could be viewed as an EE for stroke patients. Social interaction with other patients as well as with nurses and other staff would lead to more stimulation in that environment than what the patient may receive in therapy alone. Considering the physical strength and accompanying stress or cognitive issues, it is quite important to find the optimal amount of exposure to rehabilitation in clinical and experimental settings that results in improved outcomes without the deleterious side effects of excessive treatment. Our study demonstrated that 4-h enrichment was capable of producing behavioral benefit compared to the standard condition. However, further study with multiple intervention times is needed to pursue the optimal duration of EE.

### Angiogenesis After Stroke

During the long period of repair and restoration following ischemia/reperfusion injury, it is non-negligible that enhanced angiogenesis plays an important role in improving the neurological outcome. In I/R animal models, endothelial cell proliferation occurs 12–24 h after MCAO (Hayashi et al., 2003). Capillary sprouting and new vessel growth have been reported to continue for at least 3 weeks in the penumbra (Zhang et al., 2002; Hayashi et al., 2003). In ischemic stroke patients, Krupinski et al. (1994) reported that angiogenic activity occurred at 3–4 days after stroke. They also noticed that high levels of new vessel formation following stroke were correlated with better functional recovery and prolonged survival. During the process of angiogenesis, new capillaries are formed through direct proliferation and migration of endothelial progenitor cells from pre-existing blood vessels (Wang et al., 2012). Previous findings revealed that the area of CD31-immunopositive cells was significantly increased around the infarct after 28 days of treadmill training (Matsuda et al., 2011). It was also reported that physical exercise might improve motor performance and obviously increase the number of neogenetic microvessels around the penumbra following ischemic stroke, which might be related to increased CD31 expression (Hu et al., 2010; Yang et al., 2012). In the present study, we found that the PE, SE and PSE groups exhibited significantly increased MVD compared with that of the IS group following 14 days of intervention. The PSE condition increased MVD to a greater extent than the PE and SE conditions.

To further identify the possible mechanism underlying post-stroke angiogenesis, the expression levels of some angiogenic factors were examined. VEGF plays important roles in promoting the formation of primitive tubular structures as well as neuroprotection following ischemic injury (Hermann and Zechariah, 2009; Ma et al., 2012). Whereas VEGF works at the acute stage after focal cerebral ischemia, Ang-1/Tie-2 exert their functions at later stages of vascular development by mediating the interactions of endothelial cells and pericytes (Thurston et al., 1999) and promoting the maturation and stabilization of new vessels due to the formation of endothelial tight junctions and the recruitment of pericytes (Yancopoulos et al., 2000). Many reports have suggested that physical exercise and EE induced cerebral changes in the expression of angiogenic factors, such as VEGF/Ang-1 expression, which likely promote endothelial cell survival, proliferation, migration and tube formation (Seo et al., 2013; Gao et al., 2014). Social interaction could improve quality of life and decreases mortality after stroke (Boden-Albala et al., 2005). SE could decrease infarct size and brain atrophy, and increase neurogenesis and long-term functional recovery after ischemic stroke by facilitating social interaction (Fowler et al., 2002; Venna et al., 2014; Meng et al., 2015). However, the mechanisms underlying the promoting effects of SE on stroke rehabilitation is poorly understood. Our research showed that SE improved functional recovery and angiogenesis through the elevation of the expression of VEGF and Ang1/Tie 2. In the present study, we observed that cerebral ischemia-reperfusion induced angiogenic factor expression in the penumbra of MCAO rats, which showed that the PE, SE, and PSE conditions significantly upregulated angiogenic factor expression. The levation of these angiogenic factors could be due to a sequential process of increased metabolic demand associated with environments with different conditions. In addition, these findings are in line with the MVD observed in this study. Therefore, one possible mechanism for the EE-mediated enhancement of angiogenesis after stroke may be related to the upregulation of VEGF/Ang-1 expression. Some studies have shown that exposure to EE promoted angiogenesis after MCAO may be mediated by blood-borne factors such as hepatocyte growth factor (Xie et al., 2019). Other studies suggested that EE enhanced post-stroke angiogenesis *via* astrocytic HMGB1/IL-6 signaling pathway (Chen et al., 2017). There were also evidences that EE induced angiogenesis *via* regulating PI3K/AKT/GSK-3/β-catenin signaling pathway and activation of the intrinsic axonal guidance molecules in animal models of ischemic stroke (Zhan et al., 2020). However, the mechanism by which EE enhances angiogenesis after stroke needs further research.

The EEal conditions conclusively demonstrated more advantages in promoting functional recovery than no enrichment, but only exposure to the PSE condition, which contains all the components of a typical EE, yields the optimal benefits. Promoting angiogenesis may be one of the mechanisms underlying the neuroprotective effects of EEs. The findings of this study may have important translational relevance for clinical rehabilitation.

## Data Availability Statement

The original contributions presented in the study are included in the article/supplementary material, further inquiries can be directed to the corresponding author/s.

## Ethics Statement

The animal study was reviewed and approved by the Institutional Animal Care and Use Committee of Wuhan University (approval number: WP2020-08059).

## Author Contributions

XZ designed the study plan and carried out the tissue sampling, processing, and image acquisition, and drafted and edited the manuscript. JY-L provided technical support and participated in the discussion of the results. WJ-L and XP-C evaluated and revised the study design, supervised the execution of the research, and revised the manuscript. All authors contributed to the article and approved the submitted version.

## Conflict of Interest

The authors declare that the research was conducted in the absence of any commercial or financial relationships that could be construed as a potential conflict of interest.
